# The Impact of Artificial Intelligence on Internal Medicine Physicians: A Survey of Procedural and Non-procedural Specialties

**DOI:** 10.7759/cureus.69121

**Published:** 2024-09-10

**Authors:** Masab A Mansoor, Andrew F Ibrahim, Nicholas Kidd

**Affiliations:** 1 Internal Medicine, Edward Via College of Osteopathic Medicine, Monroe, USA; 2 School of Medicine, Texas Tech University Health Sciences Center, Lubbock, USA; 3 Family Medicine, University of Virginia, Charlottesville, USA

**Keywords:** artificial intelligence in medicine, artificial intelligence in telemedicine, inpatient and outpatient internal medicine, newer technology in healthcare, survey research

## Abstract

Background: Artificial intelligence (AI) is increasingly being integrated into various aspects of healthcare, including internal medicine. However, the impact of AI on physicians across different internal medicine specialties remains unclear. This study assesses AI's adoption, utilization, and perceived impact among procedural and non-procedural internal medicine physicians.

Methods: A comprehensive survey questionnaire was designed to cover current AI use, perceived impact on diagnostic accuracy, treatment decisions, patient outcomes, challenges, ethical concerns, and future expectations. The survey was distributed to a diverse sample of internal medicine physicians across various specialties, including procedural (e.g., interventional cardiology, gastroenterology) and non-procedural (e.g., endocrinology, rheumatology) fields. Responses were analyzed using descriptive statistics, chi-square tests, t-tests, and logistic regression.

Results: The survey received responses from 22 internal medicine physicians, with 64% (n=14) representing procedural specialties and 36% (n=8) representing non-procedural specialties. Sixty-eight percent (n=15) of respondents reported using AI tools in their practice, with higher adoption rates among procedural specialties (n=11, 79%) compared to non-procedural specialties (n=4, 50%). Surveyed physicians reported that AI improved diagnostic accuracy (n=12, 80%), treatment decisions (n=10, 67%), and patient outcomes (n=13, 87%). However, 55% (n=12) of respondents expressed concerns about the interpretability and transparency of AI algorithms. Non-procedural specialists were more likely to perceive AI as a threat to their job security (n=3, 38%) than procedural specialists (n=3, 21%). The most common challenges to AI adoption were lack of training (n=16, 73%), cost (n=13, 59%), and data privacy concerns (n=11, 50%).

Conclusion: This study assesses the perceived impact of AI on internal medicine physicians, highlighting the differences between procedural and non-procedural specialties. The findings underscore the need for specialty-specific considerations in developing and implementing AI tools. While AI can potentially improve diagnostic accuracy, treatment decisions, and patient outcomes, addressing challenges such as lack of training, cost, and data privacy concerns is crucial for widespread adoption. Moreover, the study emphasizes the importance of ensuring the interpretability and transparency of AI algorithms to foster trust among physicians. As AI continues to evolve, it is essential to engage internal medicine physicians across specialties in the development process to create AI tools that effectively complement their expertise and improve patient care. Further research should focus on developing best practices for AI integration in internal medicine and evaluating the long-term impact on patient outcomes and healthcare systems.

## Introduction

Artificial intelligence (AI) has emerged as a transformative force in healthcare, potentially revolutionizing various aspects of medical practice, including diagnostics, treatment decisions, and patient care [1]. In recent years, the application of AI in healthcare has gained significant momentum, with numerous studies demonstrating its potential to improve clinical outcomes, reduce costs, and enhance patient experiences [2,3].

In internal medicine, AI has shown promise in various domains, such as disease diagnosis, risk prediction, and personalized treatment planning [4]. However, the impact of AI on internal medicine physicians across different specialties remains understudied. While some studies have explored the use of AI in specific internal medicine specialties, such as cardiology [5] and gastroenterology [6], a comprehensive assessment of AI's impact on both procedural and non-procedural specialties is lacking.

Procedural specialties, such as interventional cardiology and gastroenterology, involve performing invasive procedures in addition to diagnostic and treatment tasks. These specialties may benefit from AI-assisted image analysis, real-time guidance during procedures [7], and AI-supported clinical decision-making and treatment optimization, similar to non-procedural specialties. On the other hand, non-procedural specialties, such as endocrinology and rheumatology, primarily rely on clinical decision-making and may utilize AI for diagnostic support and treatment optimization [8]. While both procedural and non-procedural specialties share typical applications of AI in diagnostic and treatment processes, understanding the differences in AI adoption, perceived impact, and challenges between these two categories of specialties is crucial for developing tailored AI solutions and implementation strategies that cater to their specific needs and workflows.

Therefore, this study aims to bridge the gap in understanding the impact of AI on internal medicine physicians by conducting a comprehensive survey across procedural and non-procedural specialties. This study seeks to provide valuable insights into the specialty-specific considerations for AI integration in internal medicine practice by assessing the current state of AI adoption, perceived benefits, challenges, and future expectations.

## Materials and methods

Study design and participants

A cross-sectional study was conducted using an online survey questionnaire, displayed in Table 5 in the Appendices. The target population included internal medicine physicians from various specialties, including procedural and non-procedural fields. Table 1 displays the demographic and professional characteristics of the study participants. Procedural specialties included interventional cardiology, gastroenterology, and pulmonology, while non-procedural specialties included endocrinology, rheumatology, and nephrology. Participants were recruited through professional medical organizations, social media platforms, and email invitations. The inclusion criteria were: (1) currently practicing as an internal medicine physician in the United States and (2) belonging to either a procedural or non-procedural specialty.

**Table 1:** Demographic and professional characteristics of the study participants

Characteristic	Total (N=22)	Procedural (n=14)	Non-procedural (n=8)
Age (SD)	45.6 (10.2)	44.9 (9.8)	46.8 (11.3)
Gender, n (%)			
- Male	14 (63.6%)	9 (64.3%)	5 (62.5%)
- Female	8 (36.4%)	5 (35.7%)	3 (37.5%)
Years in practice, mean (SD)	15.2 (8.6)	14.6 (8.1)	16.3 (9.7)
Practice setting, n (%)			
- Academic medical center	9 (40.9%)	5 (35.7%)	4 (50.0%)
- Community hospital	8 (36.4%)	6 (42.9%)	2 (25.0%)
- Private practice	5 (22.7%)	3 (21.4%)	2 (25.0%)
Specialty, n (%)			
- Interventional cardiology	6 (27.3%)	6 (42.9%)	-
- Gastroenterology	5 (22.7%)	5 (35.7%)	-
- Pulmonology	3 (13.6%)	3 (21.4%)	-
- Endocrinology	3 (13.6%)	-	3 (37.5%)
- Rheumatology	3 (13.6%)	-	3 (37.5%)
- Nephrology	2 (9.1%)	-	2 (25.0%)

The research team developed the survey questionnaire based on a review of existing literature and expert consultations. The questionnaire consisted of four main sections: (1) demographic and professional characteristics, (2) current use of AI tools in practice, (3) perceived impact of AI on various aspects of patient care, and (4) challenges and concerns related to AI adoption. The survey included multiple-choice and open-ended questions to capture quantitative and qualitative data. The questionnaire was pilot-tested with a small group of internal medicine physicians to ensure clarity and relevance.

Validation

Several steps were taken during the questionnaire development and validation process to establish the survey's validity and reliability. First, content validity was established by developing the study based on a comprehensive literature review and expert consultations with a panel of internal medicine physicians representing procedural and non-procedural specialties. The panel reviewed the questionnaire for relevance, clarity, and comprehensiveness, and their feedback was incorporated to refine the questions. Second, face validity was assessed by pilot-testing the revised questionnaire with a small group of internal medicine physicians from various specialties to evaluate its clarity, relevance, and ease of completion. Their feedback was used to make final adjustments to the questionnaire. Third, internal consistency reliability was assessed by calculating Cronbach's alpha for survey items measuring similar constructs, with values found to be within the acceptable range (α > 0.7). Finally, cross-specialty reliability was ensured by comparing responses from procedural and non-procedural specialties using chi-square tests for categorical variables and t-tests for continuous variables, with non-significant differences in the demographic and professional characteristics of the two groups, suggesting the survey's reliability across specialties.

Data collection

The survey was administered electronically using a secure online platform. Participants were provided with an informed consent form and assured of the confidentiality of their responses. The survey remained open for three months, with reminder emails sent to non-respondents every two weeks. Participation was voluntary, and no incentives were offered.

It is important to note that the electronic distribution of the survey may have introduced a potential selection bias, as it might have favored physicians who are more technologically savvy and comfortable with online platforms. This bias could have led to an overrepresentation of physicians with a greater interest in or experience with AI, potentially influencing the study results.

Data analysis

Descriptive statistics, including frequencies, percentages, means, and standard deviations, were used to summarize the demographic and professional characteristics of the participants, as well as their responses to the survey questions. Chi-square tests were employed to compare categorical variables between procedural and non-procedural specialties, while independent t-tests were used for continuous variables. Logistic regression analysis was performed to identify factors associated with AI adoption and perceived impact, with specialty type (procedural vs. non-procedural) as the primary independent variable. Qualitative data from open-ended questions were analyzed using thematic analysis to identify common themes and patterns.

Given the exploratory nature of this study and the small sample size, we primarily used descriptive statistics. We used Fisher's exact test for categorical variables to compare procedural and non-procedural specialties due to the small sample size. We used the Mann-Whitney U test for continuous variables as the data did not meet assumptions for parametric tests. All statistical analyses were conducted using SPSS version 26.0 (IBM Corp., Armonk, NY, USA), with a p-value of <0.05 considered statistically significant.

Ethical considerations

The Ethics Committee of Mansoor Pediatrics approved this study and determined that this study falls under IRB exemption status §46.104(3)(i)(A-C) regarding exempt research. All participants provided informed consent before completing the survey. No personally identifiable information was collected, and the data were stored securely in compliance with data protection regulations.

## Results

Survey response and participant characteristics

Twenty-two internal medicine physicians completed the survey, representing a response rate of 65% (22/34). Among the respondents, 14 (64%) were from procedural specialties, and eight (36%) were from non-procedural specialties. Table 1 summarizes the demographic and professional characteristics of the participants. Qualitative analysis of open-ended responses revealed several recurring themes. For example, one procedural specialist noted: "AI has significantly improved our ability to detect subtle abnormalities in imaging studies, leading to earlier diagnoses." Conversely, a non-procedural specialist expressed concerns: "While AI tools can process vast amounts of data quickly, I worry about over-reliance leading to atrophy of clinical reasoning skills."

Current use of AI tools in practice

Table 2 displays that 68% (n=15) of the respondents reported using AI tools in their practice. The adoption rate was higher among procedural specialties (n=11, 79%) than non-procedural specialties (n=4, 50%). The most commonly used AI applications were clinical decision support systems (n=12, 80%), image analysis tools (n=9, 60%), and predictive modeling for risk stratification (n=7, 47%).

**Table 2:** Current use of artificial intelligence (AI) tools in practice

AI application	Total (N=15)	Procedural (n=11)	Non-procedural (n=4)	P-value
Clinical decision support systems	12 (80%)	9 (82%)	3 (75%)	0.76
Image analysis tools	9 (60%)	8 (73%)	1 (25%)	0.10
Predictive modeling for risk stratification	7 (47%)	6 (55%)	1 (25%)	0.31

Specific examples of clinical decision support systems include AI-powered diagnostic tools that analyze patient data, such as symptoms, medical history, and test results, to suggest potential diagnoses and treatment options, like systems that use machine learning algorithms to assist physicians in diagnosing rare genetic disorders based on patient data and clinical presentation. Additionally, AI-based tools can provide personalized medication recommendations by considering patient factors, such as age, comorbidities, and potential drug interactions, helping physicians optimize treatment plans and reduce the risk of adverse drug events.

Examples of image analysis tools include AI algorithms that analyze medical images, such as X-rays, CT scans, or MRIs, to detect abnormalities and assist in diagnosis. For example, systems can identify pulmonary nodules on chest X-rays, aiding in the early detection of lung cancer. Moreover, AI-powered tools can analyze endoscopic images to detect and classify gastrointestinal lesions, such as polyps or ulcers, assisting gastroenterologists in making more accurate diagnoses during procedures.

Predictive modeling for risk stratification includes AI models that predict a patient's risk of developing certain conditions, such as cardiovascular disease or diabetes, based on their medical history, lifestyle factors, and genetic data, helping physicians identify high-risk patients and implement preventive measures. Additionally, AI algorithms can predict the likelihood of hospital readmission or mortality in patients with chronic conditions, such as heart failure or chronic obstructive pulmonary disease (COPD), enabling physicians to provide more targeted interventions and follow-up care.

Perceived impact of AI on patient care

As outlined in Table 3, the majority of the respondents believed that AI had a positive impact on various aspects of patient care. Specifically, 80% (n=12) reported that AI improved diagnostic accuracy, 67% (n=10) indicated that AI enhanced treatment decisions, and 87% (n=13) perceived that AI led to better patient outcomes. However, 55% (n=12) of the respondents expressed concerns about the interpretability and transparency of AI algorithms.

**Table 3:** Perceived impact of artificial intelligence (AI) on patient care

Aspect of patient care	Total (N=15)	Procedural (n=11)	Non-procedural (n=4)	P-value
Diagnostic accuracy	12 (80%)	9 (82%)	3 (75%)	0.76
Treatment decisions	10 (67%)	8 (73%)	2 (50%)	0.40
Patient outcomes	13 (87%)	10 (91%)	3 (75%)	0.42
Algorithm interpretability	12 (55%)	7 (64%)	5 (63%)	0.96

Challenges and concerns related to AI adoption

As noted in Table 4, respondents identified the most common challenges to AI adoption as lack of training (n=16, 73%), cost (n=13, 59%), and data privacy concerns (n=11, 50%). Non-procedural specialists were more likely to perceive AI as a threat to their job security (n=3, 38%) than procedural specialists (n=3, 21%).

**Table 4:** Challenges and concerns related to artificial intelligence (AI) adoption

Challenge/Concern	Total (N=22)	Procedural (n=14)	Non-procedural (n=8)	P-value
Lack of training	16 (73%)	10 (71%)	6 (75%)	0.85
Cost	13 (59%)	8 (57%)	5 (63%)	0.80
Data privacy concerns	11 (50%)	7 (50%)	4 (50%)	1.00
Threat to job security	6 (27%)	3 (21%)	3 (38%)	0.41

## Discussion

This study provides insights into the current state of AI adoption and its perceived impact on internal medicine physicians across procedural and non-procedural specialties. The findings highlight the growing utilization of AI tools in clinical practice, with a higher adoption rate among procedural specialties. This trend is consistent with previous studies that have demonstrated the successful integration of AI in procedural fields such as radiology and pathology [9,10].

Most respondents perceived AI as positively impacting various aspects of patient care, including diagnostic accuracy, treatment decisions, and patient outcomes. These results align with the growing body of literature supporting the potential of AI to enhance clinical decision-making and improve patient care [11,12]. However, the concerns expressed by the respondents regarding the interpretability and transparency of AI algorithms underscore the need for developing explainable AI models that can foster trust among physicians and patients [13].

A larger percentage of non-procedural specialists (n=3, 38%) reported perceiving AI as threatening their job security than procedural specialists (n=3, 21%). However, this finding was not statistically significant (p=0.41), suggesting that concerns about AI's impact on job security may be shared across internal medicine specialties, regardless of their procedural nature. The higher adoption rate of AI tools among procedural specialties (79% vs 50%) may be attributed to the frequent use of imaging and diagnostic procedures in these fields. For instance, AI-assisted image analysis tools have been rapidly integrated into specialties like interventional cardiology and gastroenterology. On the other hand, non-procedural specialties may rely more on clinical judgment and patient history, areas where AI integration is still evolving. It is possible that the relatively small sample size in our study limited our ability to detect significant differences between the two groups.

Additionally, the lack of a significant difference may reflect the early stage of AI integration in internal medicine, where the potential impact on job security is not yet fully realized or differentiated between specialties. As AI technologies continue to advance and become more widely adopted, future studies with larger sample sizes should revisit this question to assess the evolving impact of AI on job security across different internal medicine specialties [14]. As AI advances in diagnosis and treatment planning tasks, non-procedural specialists may feel more vulnerable to the potential displacement of their roles. This highlights the importance of addressing AI's ethical and social implications in healthcare and ensuring that AI is developed and implemented in a way that augments, rather than replaces, human expertise [15].

The challenges identified by the respondents, such as lack of training, cost, and data privacy concerns, are consistent with the barriers to AI adoption reported in previous studies [16,17]. These findings emphasize the need for dedicated training programs to equip physicians with the necessary skills to utilize AI tools in their practice effectively. Furthermore, addressing the cost and data privacy concerns by developing affordable and secure AI solutions is crucial for widespread adoption [18]. 

Limitations

This study has several limitations. First, the small sample size and the focus on a single institution may limit the generalizability of the findings. Future studies should include a more extensive and diverse sample of internal medicine physicians from multiple institutions. Second, the survey relied on self-reported data, which may be subject to recall and social desirability biases. 

It is important to note the several factors that could contribute to non-significant findings. First, the study's small sample size (n=22) may have limited the statistical power to detect significant differences between the two groups. Future research with larger sample sizes is needed to investigate further potential specialty-specific differences in AI adoption and its impact on internal medicine physicians. Second, the lack of significant differences may suggest that the challenges and concerns related to AI implementation are shared across specialties, regardless of their procedural nature, highlighting the need for broader, specialty-agnostic strategies to address the barriers to AI adoption in healthcare, such as providing adequate training, ensuring data privacy, and developing interpretable AI algorithms [18]. Finally, the non-significant findings may reflect the early stage of AI integration in internal medicine, where the impact of AI on job security and other aspects of clinical practice may not yet be fully realized or differentiated between specialties. As AI technologies continue to advance and become more widely adopted, future studies should revisit these questions to assess the evolving impact of AI on different internal medicine specialties. 

The type of electronic medical record (EMR) system used by different specialists could indeed influence AI adoption rates. Some EMR systems have built-in AI capabilities or easier integration with third-party AI tools. Future research should explore the relationship between EMR systems and AI adoption across internal medicine specialties. Another potential limitation is the possibility of selection bias introduced by the electronic survey distribution method. As the survey was administered online, it may have favored the participation of physicians who are more technologically savvy and comfortable with online platforms. This bias could have led to an overrepresentation of physicians with a greater interest in or experience with AI, potentially influencing the study results. Future studies should consider using a mix of survey distribution methods, such as paper-based surveys or in-person interviews, to mitigate this potential bias and ensure a more representative sample of internal medicine physicians.

Future research

Future research should explore these differences in depth to develop tailored strategies for AI integration across various internal medicine specialties [18]. Objective measures of AI adoption and impact should be considered in future research. To address the limitations of relying solely on self-reported data, future research should consider incorporating objective measures to gain a more comprehensive understanding of the impact of AI on internal medicine physicians and patient care. This strategy can include analyzing electronic health record data to assess the real-world impact of AI on clinical decision-making and patient outcomes, collecting objective clinical outcomes data through chart reviews or clinical trials, and measuring workflow and efficiency metrics using time-motion studies or workflow analysis tools.

Additionally, evaluating the performance of AI algorithms using standardized datasets and assessing physician competence and confidence through objective tests or simulations can provide valuable insights. Economic data, such as costs associated with AI implementation and potential cost savings, can also offer objective measures of the financial impact of AI in healthcare settings [18]. By incorporating these objective measures in future research, investigators can triangulate findings from self-reported data and strengthen the validity and generalizability of the findings, ultimately providing a more robust evidence base for understanding the impact of AI in healthcare.

## Conclusions

This study assesses the impact of AI on internal medicine physicians, highlighting the differences between procedural and non-procedural specialties. The findings demonstrate the growing adoption of AI tools in clinical practice, with procedural specialties reporting higher utilization rates. While most respondents perceived AI as having a positive impact on patient care, concerns regarding the interpretability and transparency of AI algorithms were prevalent. These findings underscore the importance of considering specialty-specific factors when developing and implementing AI solutions in healthcare.

The challenges identified, such as lack of training, cost, and data privacy concerns, emphasize the need for dedicated efforts to overcome these barriers. As AI continues to evolve and transform healthcare, it is essential to engage internal medicine physicians across specialties in the development and implementation process. We can create AI tools that complement human expertise and improve patient outcomes by fostering collaboration between AI developers and clinicians. This study provides insight into the current state of AI adoption and its perceived impact on internal medicine physicians. The findings highlight the need to address the challenges and concerns of AI implementation and emphasize the importance of considering specialty-specific factors in developing and deploying AI solutions.

## Appendices

**Table 5:** Survey questionnaire

Section	Question
Demographic and Professional Characteristics	1. What is your age?
	2. What is your gender? a) Male b) Female c) Other d) Prefer not to answer
	3. How many years have you been practicing medicine?
	4. What is your primary practice setting? a) Academic medical center b) Community hospital c) Private practice d) Other (please specify)
	5. What is your specialty? a) Interventional cardiology b) Gastroenterology c) Pulmonology d) Endocrinology e) Rheumatology f) Nephrology
Current Use of AI Tools in Practice	6. Do you currently use any AI tools in your practice? a) Yes b) No
	7. If yes, which AI applications do you use? (select all that apply) a) Clinical decision support systems b) Image analysis tools c) Predictive modeling for risk stratification d) Other (please specify)
Perceived Impact of AI on Patient Care	8. Do you believe AI has improved diagnostic accuracy in your practice? a) Yes b) No c) Unsure
	9. Do you believe AI has enhanced treatment decisions in your practice? a) Yes b) No c) Unsure
	10. Do you believe AI has led to better patient outcomes in your practice? a) Yes b) No c) Unsure
	11. Are you concerned about the interpretability and transparency of AI algorithms? a) Yes b) No c) Unsure
Challenges and Concerns Related to AI Adoption	12. What are your main challenges in adopting AI tools in your practice? (select all that apply) a) Lack of training b) Cost c) Data privacy concerns d) Other (please specify)
	13. Do you perceive AI as a threat to your job security a) Yes b) No c) Unsure
Open-ended Questions	14. What do you believe are the most significant benefits of AI in internal medicine?
	15. What do you believe are the most significant risks or drawbacks of AI in internal medicine?
	16. How do you think AI will impact the role of internal medicine physicians in the future?
	17. What resources or support would you need to integrate AI into your practice effectively?
	18. Do you have any additional comments or insights regarding AI in internal medicine?

## References

[1] Topol EJ (2019). High-performance medicine: the convergence of human and artificial intelligence. Nat Med.

[2] Rajkomar A, Dean J, Kohane I (2019). Machine learning in medicine. N Engl J Med.

[3] Ngiam KY, Khor IW (2019). Big data and machine learning algorithms for health-care delivery. Lancet Oncol.

[4] Gulshan V, Peng L, Coram M (2016). Development and validation of a deep learning algorithm for detection of diabetic retinopathy in retinal fundus photographs. JAMA.

[5] Johnson KW, Torres Soto J, Glicksberg BS (2018). Artificial intelligence in cardiology. J Am Coll Cardiol.

[6] Le Berre C, Sandborn WJ, Aridhi S (2020). Application of artificial intelligence to gastroenterology and hepatology. Gastroenterology.

[7] Sarwar S, Dent A, Faust K, Richer M, Djuric U, Van Ommeren R, Diamandis P (2019). Physician perspectives on integration of artificial intelligence into diagnostic pathology. NPJ Digit Med.

[8] Briganti G, Le Moine O (2020). Artificial intelligence in medicine: today and tomorrow. Front Med (Lausanne).

[9] Hosny A, Parmar C, Quackenbush J, Schwartz LH, Aerts HJ (2018). Artificial intelligence in radiology. Nat Rev Cancer.

[10] Bera K, Schalper KA, Rimm DL, Velcheti V, Madabhushi A (2019). Artificial intelligence in digital pathology - new tools for diagnosis and precision oncology. Nat Rev Clin Oncol.

[11] Adadi A, Berrada M (2018). Peeking inside the black-box: a survey on explainable artificial intelligence (XAI). IEEE Access.

[12] Verghese A, Shah NH, Harrington RA (2018). What this computer needs is a physician: humanism and artificial intelligence. JAMA.

[13] Char DS, Shah NH, Magnus D (2018). Implementing machine learning in health care - addressing ethical challenges. N Engl J Med.

[14] Kelly CJ, Karthikesalingam A, Suleyman M, Corrado G, King D (2019). Key challenges for delivering clinical impact with artificial intelligence. BMC Med.

[15] Recht MP, Dewey M, Dreyer K, Langlotz C, Niessen W, Prainsack B, Smith JJ (2020). Integrating artificial intelligence into the clinical practice of radiology: challenges and recommendations. Eur Radiol.

[16] Panch T, Mattie H, Celi LA (2019). The "inconvenient truth" about AI in healthcare. NPJ Digit Med.

[17] Davenport T, Kalakota R (2019). The potential for artificial intelligence in healthcare. Future Healthc J.

[18] Sebastian AM, Peter D (2022). Artificial intelligence in cancer research: trends, challenges and future directions. Life (Basel).

